# Barriers and facilitators to adoption, implementation and sustainment of obesity prevention interventions in schoolchildren– a DEDIPAC case study

**DOI:** 10.1186/s12889-018-6368-7

**Published:** 2019-02-15

**Authors:** C. B. Hayes, M. P. O’Shea, C. Foley-Nolan, M. McCarthy, J. M. Harrington

**Affiliations:** 1 Public Health and Primary Care, Institute of Population Health, School of Medicine, Trinity College Dublin, Dublin, Ireland; 2 Safefood and School of Public Health, Western Gateway Building, University College Cork, Cork, Ireland; 3 Cork University Business School, University College Cork, Cork, Ireland; 4 School of Public Health, Western Gateway Building, University College Cork, Cork, Ireland

**Keywords:** Implementation barriers, Facilitators, School-based, Obesity prevention

## Abstract

**Background:**

The aim of the study was to explore the implementation of school based diet and physical activity interventions with respect to the barriers and facilitators to adoption, implementation and sustainability; supportive actions required for implementation and recommendations to overcome identified barriers. Two interventions rolled out nationally in Ireland were chosen; Food Dudes, a programme to encourage primary school children to consume more fruit and vegetables and Green Schools Travel (GST), an active travel to school programme in primary and secondary schools. Trained school coordinators (teachers) cascade the programmes to other teaching staff.

**Methods:**

Multiple case study design using qualitative semi-structured interviews with key stakeholders: primary and secondary school teachers, school coordinators, project coordinators/managers, funders and intermediaries. Fifteen interviews were conducted. Data were coded using a common categorization matrix. Thematic analysis was undertaken using the Adoption, Implementation and Maintenance elements of the RE-AIM implementation framework.

**Results:**

Good working relationships within and across government departments, intermediaries and schools were critical for intervention adoption, successful implementation and sustainability. Organisational and leadership ability of coordinators were essential. Provision of participation incentives acted as motivators to engage children’s interest. A deep understanding of the lives of the target children was an important contextual factor. The importance of adaptation without compromising core components in enhancing intervention sustainability emerged. Successful implementation was hindered by: funding insecurity, school timetable constraints, broad rather than specific intervention core components, and lack of agreement on conduct of programme evaluation. Supportive actions for maintenance included ongoing political support, secure funding and pre-existing healthy lifestyle policies.

**Conclusions:**

Successful implementation and scale up of public health anti-obesity interventions in schools is dependent on good contextual fit, engagement and leadership at multiple levels and secure funding. Recommendations to overcome barriers include: capacity to deliver within an already overcrowded curriculum and clear specification of intervention components within a conceptual framework to facilitate evaluation. Our findings are generalisable across different contexts and are highly relevant to those involved in the development or adaptation, organisation or execution of national public health interventions: policy makers, guidelines developers, and staff involved in local organisation and delivery.

**Electronic supplementary material:**

The online version of this article (10.1186/s12889-018-6368-7) contains supplementary material, which is available to authorized users.

## Background

Obesity is the single biggest global issue affecting health in developed countries and a key challenge in meeting the United Nations sustainable development goal of achieving good health and well-being [1]. It is increasingly recognised that a multi-faceted systems approach is needed to address complex persistent “wicked” public health problems like obesity [2]. One aspect of this systems approach is to employ population wide policies and interventions to address obesity prevention in the various ‘settings’. The school provides a unique captive setting for implementation and scale up of population based approaches to prevention of childhood overweight and obesity. Childhood obesity is a significant precursor of adult obesity [3–7]. However, positive health behaviours if embedded in childhood are more likely to persist into adulthood. Within the school setting single or multi-component interventions that address dietary intake, and/or physical activity or sedentary behavior have shown limited effectiveness in reducing sedentary behavior and BMI [8–10].

There is empirical evidence to suggest that successful outcomes of public health interventions depend not only on the existence of the specific intervention components needed to achieve behaviour change [11] but also on the extent to which they are implemented in the real world setting [12]. A greater understanding of context and how and to what extent interventions are implemented to ensure system-wide sustainability is required. An overarching synthesis of the empirical evidence [13] identified 83 conditions to be important for successful implementation of interventions and policies promoting a healthy diet, physical activity (PA), and a reduction in sedentary behaviour using the five domains of the Reach, Effectiveness, Adoption, Implementation and Maintenance (RE-AIM) evaluation framework [14, 15].

Within the school setting a meta-synthesis of 18 qualitative studies, which explored the views of parents, school staff and students on the overall role of the primary school in preventing childhood obesity, concurred that the school is an important setting for obesity prevention, in promoting and providing opportunities for healthy eating and physical activity and the need for schools to work with parents [14].

Implementation strategies are defined as “methods or techniques used to enhance the adoption, implementation, and sustainability of a clinical program or practice” [15]. There is limited in-depth research to date on the implementation strategies necessary for successful adoption, implementation and sustainment for scale-up of public health interventions addressing diet and or physical activity particularly within the primary school setting. Some school-based research carried out in older children has identified general themes e.g. commitment and leadership within schools as enabling strategies and a lack of time and external resources as barriers [16–19],

The focus of the current study was, therefore, to qualitatively explore the implementation of school-based interventions targeting childhood obesity. The study was part of a larger series of European case studies conducted by the Determinants of Diet and Physical Activity Knowledge Hub, DEDIPAC-KH, the first research project of the European Union’s (EU) Joint Programming Initiative - Healthy Diet for a Healthy Life (JPI-HDHL), which aimed to provide better insight into the determinants of diet, physical activity and sedentary behaviour across the life course [20].

## Methods

The overall aim of this study was to explore and categorise factors likely to result in successful implementation and transferability of multi-component dietary, physical activity or sedentary behaviour interventions in Irish schools and those factors that hinder successful implementation. It sought to provide insight from the stakeholders’ viewpoint into the supportive actions required for successful implementation and how barriers to implementation might be overcome. The Adoption, Implementation and Maintenance elements of the RE-AIM (Reach, Effectiveness, Adoption, Implementation, Maintenance) implementation framework, used extensively for evaluating implementation of public health interventions [21] was used to categorise the findings.

### Study design and case selection

A case study is an empirical method aimed at investigating contemporary phenomena in their context and in particular where the boundary between the phenomenon and its context may be unclear [22]. Hence a multiple case study design was deemed most appropriate for this study.

### Intervention selection

Informal meetings with Irish collaborators and a brief scoping review of the available literature led to case selection. Two school-based multi-component interventions were chosen - The Food Dudes (FD) Healthy Eating Programme and the Travel Theme of the Green Schools Environmental Programme, which is known internationally as Eco-Schools. Both of these programmes are purposive interventions aimed at influencing positive changes in one or more factors affecting behaviour. Both programmes have been rolled out nationally in the Republic of Ireland and are subject to evaluation and/or ongoing audit [23–28].

### Intervention description

#### Food Dudes healthy eating programme

**Fig. 1:**
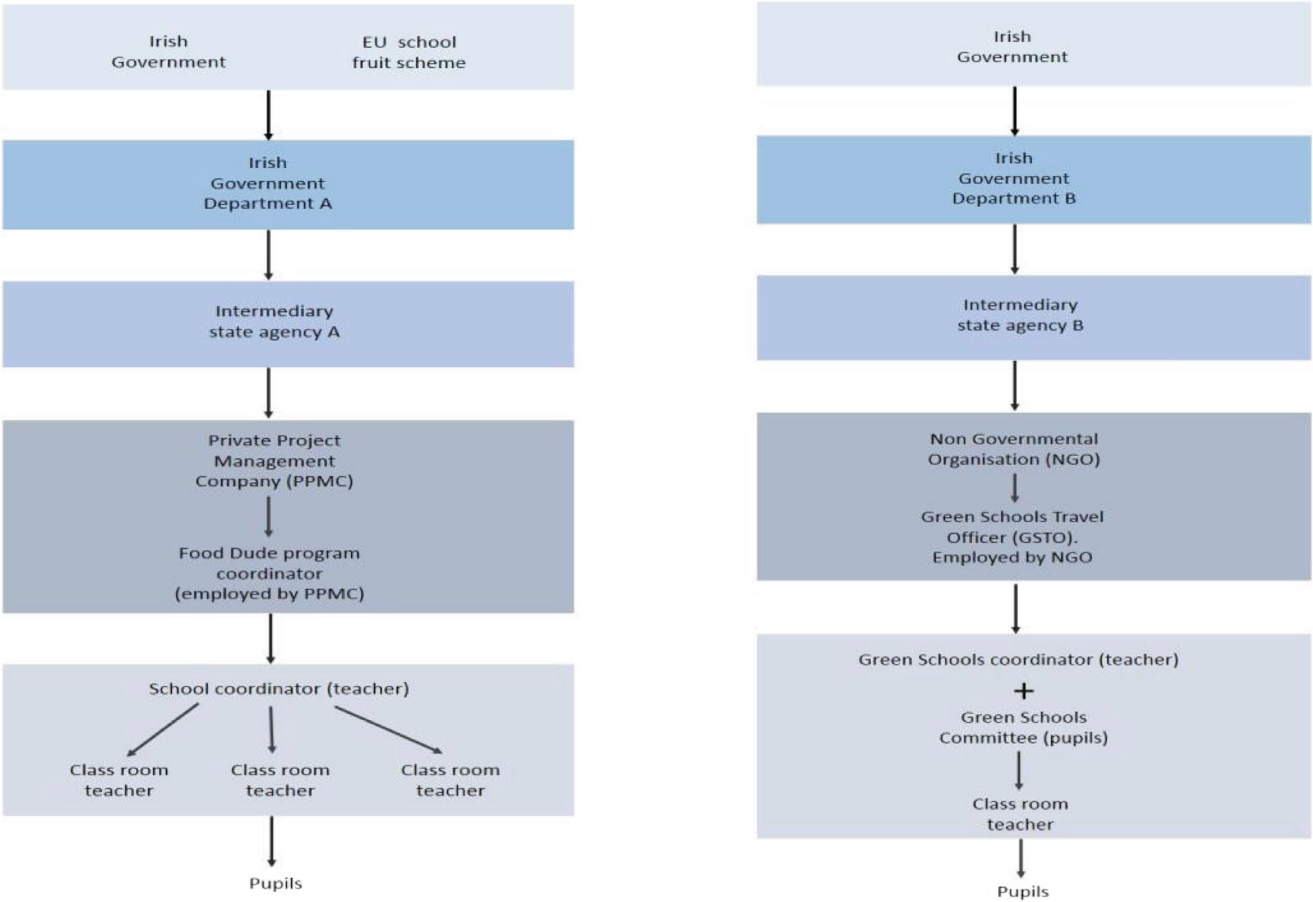
(**a**) Organisational structure Food Dudes, (**b**) Organisational structure GTS (Primary Schools)

FD is a peer modelling and rewards-based intervention to increase fruit and vegetable consumption in primary school children [25, 29]. It was developed by researchers in Bangor University Wales based on the theory that provision and repeated tasting of fruit and vegetables over a period will result in a sustainable increase in fruit and vegetable consumption. Phase 1 involves provision and repeated tasting of fruit and vegetables over a 16-day intervention period with the support of cartoon role models (Food Dudes Heroes) and small rewards, followed by a home phase where fruit and vegetables are supplied from home (Phase 2). It was first rolled out in Ireland in 2005 [28]. It is jointly funded by the Irish Government and the European Union through the School Fruit Scheme. The programme is managed nationally by the Irish Food Board (An Bord Bia) and delivered by a private project management company (PMC). The PMC is responsible for training FD coordinators (one or two teachers within each school) and the organisation of fruit and vegetable suppliers. The FD coordinators are responsible for cascading the information to other teachers and organising deliveries within the school during the intervention phase. Schools are invited to take part in the FD programme on a regional / district basis. The original Food Dudes Programme was completed in 2014 having reached 95% of all 3300 primary schools in Ireland [30]. Participation in the FD programme is voluntary. A Food Dudes ‘Boost’ programme was introduced in 2015. A flow diagram of FD is shown (Fig. 1a).

#### Green Schools Travel

Green Schools Travel (GST) is the fourth of seven themes of the Green-Schools Programme (GSP). GST aims to promote the use of sustainable modes of transport to school e.g. walking, cycling and public transport use. Following a two-year pilot in the Greater Dublin Area GST has been rolled out nationally since 2008 in schools that have completed the first three themes of the GSP (Litter and Waste, Energy and Water). GST is funded by national government via the National Transport Authority to a non-governmental organisation (NGO) who operate the programme. Between 2006 and 2011, 539/3030 primary schools (17%) and 55/700 (8%) secondary schools took part in GST [23]. Schools take two years to complete GST [23]. Schools set their own travel targets. Participation in GST is voluntary and schools self-select to participate in the programme.

A network of Green School Travel Officers (GSTOs), employed by the NGO, assists schools throughout the process. The programme is organised within the school by a designated teacher who volunteers for the role as GST coordinator and a pupil-led GST committee. The GST coordinator and committee cascade the programme to all classes within the school. A flow diagram of GST in primary schools is shown (Fig. 1b).

### Case study sites and sampling

Permission to use the selected programmes as cases was obtained from government departments and intermediaries involved in the management and/or funding of the programmes. Introductory meetings were held with agencies responsible for their organisation and management. These meetings provided background information and assisted in the purposeful identification of potential interviewees involved in implementation of the interventions. All key stakeholders were invited to participate: senior personnel from government departments, intermediary agencies (NGO and PMC) and educationalists.

Inclusion criteria for eligible schools were: school location; Greater Dublin Area (counties Dublin, Wicklow, Kildare; total population 1,709,864), [31] urban rural mix, school type; primary or secondary (GST only) and status of the intervention recently completed or completed within a given timeframe. There were 552 eligible FD and 364 GST schools. From discussion with the above stakeholders and guided by the literature [32] an initial sample of eight schools, four from each programme was deemed sufficient to provide adequate sample size to achieve data saturation, and to be feasible within the timelines and resources for the study.

The FD management company, at their request selected and contacted four schools meeting these criteria. From a list of potential GST schools provided by the NGO, four schools were randomly selected using a random numbers table. Potential participants from all stakeholder groups were sent an introductory letter, explanatory leaflet and consent form and invited to participate in face-to-face interviews. Follow up was conducted by telephone if no response was obtained within two weeks of initial contact.

### Data collection

Fifteen semi-structured in-depth interviews were conducted (Table 1). An interview topic guide developed by the European DEDIPAC research team guided the direction of interview questions. This was informed by an umbrella review of conditions identified as being associated with successful implementation of diet and/or physical activity interventions/policies [33]. The topic guide was adapted to suit the role/knowledge of the interviewee, which allowed for deductive and inductive themes to emerge. The list of questions covered in the topic guide was the same for both programmes and are included as a supplementary file (Additional file 1).

**Table 1:** Description of participants

	FD	GST	Total
Total number of interviews	7^a^	8^b^	15
Total number of participants	9	9	18
Major stakeholders (Funders, intermediaries)	4 (3 female, 1 male)	6 (5 female, 1 male)	10
School Teachers	4 (all female)	3 (all female)	7
Academic researcher	1 (male)	N/A	1

^a^ Two interviews had two participants

^b^ One interview had two participants

Fourteen interviews were conducted face-to-face and one by telephone by a post-doctoral researcher with previous experience in conducting qualitative research (MO’S), who was recruited specifically for the project and was not known to the stakeholders. One interview was observed directly by the prinicpal researcher (CH) to ensure fidelity to the topic guide. The mean interview duration was 55 min (28–69 min). All face-to-face interviews were conducted in a private office at the participant’s work place. Two initial interviews were not recorded; however, extensive notes were made following each interview. Thirteen interviews were digitally audio recorded and transcribed verbatim by an external stenographer. Participant names were removed prior to transcription. The transcripts were verified by reading a selection while listening to the audio recordings(MO’S). Any discrepancies between the transcripts and audio recordings were rectified before coding.

### Data analysis

The interviews were coded in NVivo Version 10.2 (QSR 2014), using a common categorization matrix developed by the European DEDIPAC research team [34]. The matrix was informed by the review of conditions identified as being associated with successful implementation of interventions aimed at improving diet and/or levels of physical activity [33]. Initial coding was conducted by MOS and verified by CH and discrepancies were amended following discussion to clarify coding and emergent themes. Coded data were subsequently exported into Microsoft Excel version 16.6.5 (2018 Southampton), and collated into themes (MOS). A thematic analysis of data was carried out using a hybrid inductive-deductive approach to identify patterns in the data.

Themes were then classified (CH and MOS) according to Adoption, Implementation and Maintenance (Sustainment) of the interventions as follows:

- Factors facilitating adoption, implementation and maintenance
- Factors hindering adoption, implementation and maintenance
- Supportive actions for adoption, implementation and maintenance
- Recommendations to overcome barriers to adoption, implementation and maintenance

The Consolidated criteria for reporting Qualitative Studies (COREQ-32 item checklist) were consulted in reporting the study findings [35].

## Results

### Description of interview participants

A breakdown of participants is shown in Table 1. Seven schools agreed to participate. All participants contacted in those schools agreed to be interviewed. Three interviews had two participants. There were no notable differences between male and female respondents. Data saturation was reached following completion of interviews within the initial sample of six primary schools with no new themes emerging [32]. As findings particular to secondary schools were less robust and timing and resources did not permit further sampling, these findings were excluded from this paper.

### Factors facilitating adoption, implementation and maintenance (Table 2)

#### Adoption

Good communication, collaboration and support from various government departments (e.g. Department of Transport, Department of Agriculture and Department of Education) were seen as key to developing a strategy for encouraging schools to adopt either programme. Engagement of all school partners (parents, teachers, principal and students) was found to be a critical success factor in the adoption and implementation of both programmes. The ability of the FD coordinator to provide leadership and effectively organise and delegate responsibility for the delivery of the fruit and vegetables was also important (cited*3).

**Table 2:** Quotations illustrating factors facilitating adoption, implementation and maintenance

(RE)AIM Parameters (Bold)	
**Adoption**	
Collaboration and Communication	…“*these Travel Officers and other officers in (NGO) already have an open door into these schools. They have built up a relationship”* (Civil Servant, Government Department B).
	*…“Officers around the country would come up with different things and then at our meetings we show them to each other… Then we have a shared database that we all link into”* [GSTO].
**Implementation**	
Characteristics of settings	*…“the class teachers were very, like they were really good like we just said this is all happening, this is invading your classroom…and they were like yeah grand and they all just hopped to it*” [FD School Coordinator/Teacher]
	*…“we have a lady that kind of goes around with our school would get fruit...and some sandwiches as well delivered (and)…kind of sorted that out into baskets, so she also did the em fruit and veg for us”.* [FD School Coordinator/Teacher]
Implementers characteristics	*…“it’s really about how interested the coordinator is within the school… they’re the people, the drivers within the school that really push the theme through”* [Intermediary Agency A, Management Official]. *“I also think the travel theme is one where the chidren are really, really central. It’s driven by the chidren as well, like we have to make sure tht the ideas that we’ll say we’ll do happen” [GS Coordinator].*
Implementation delivery, protocols, fidelity (including dose)	*“…There would be phone calls on day 4 and day 5 where the programme changes…so the first school days of the programme the children have to take a bite in order to be rewarded. But from day 5 through to day 16, you have to eat a portion... So there would be phone calls just to check that the coordinators would remind all teachers…There would be visit again on day 16… depending on the degree of support that the teachers needed and if they had extra queries… and then we’d have a visit approximately 4 to 6 weeks later,… for phase 2, to see how they are getting on.”* [FD PMC Official]
	*…“Like schools would have walk to school days, WoW (Walk on Wednesdays) days or CoW (Cycle on Wednesdays) days and where it’s … recorded”* [GSTO].
Adaptation and customisation	*“We have a few children that maybe just wouldn’t kind of like fruit and veg at all. And they actually did try it, because they really wanted the prize”.* [FD School coordinator/ Primary School Teacher]
	*…“with special schools it’s been very much about working with the teachers in those schools to apply the principles behind the programme, em but to match them to the needs of the children and their specific need. So even within a class in a special school there might be different children who are being rewarded for doing different things…for some if they have a terrible aversion to em to bananas or yellow foods,… it’s about edging them closer and closer to eating”. [FD PMC Official]*
	*“It’s about kind of… meshing it into this fabric of the school day…by doing it during our little break and big break and kind of doing the video then” [FD School Coordinator)*
	*“I know the paperwork has kind of come down the last couple of themes, it is more tick the box or list out which is great for the kids… because it did feel like writing essays at one stage”* [Green Schools Coordinator]
Incentives and rewards	*…“it’s about the class and about their attitude in terms of the Golden Boot (award) you know you’re putting them one step away from getting the Golden Boot. Yeah but also like they (pupils) want to (achieve it).”* [GSTO].
Process evaluation	*...“it’s hands up surveys in schools which is fine and you don’t expect young kids to lie*” [Civil Servant, Government Department B]
Simplicity of the intervention	*“We designed a system whereby the rewards would be packaged and labelled per phase and per day …it could be packed per classroom as well. So the teacher would open the box for his or her own classroom inside that would be all the phase 1 rewards, clearly packaged and labelled day 1 reward, day 2 reward, day 3 and so forth… and then the same for phase 2…”* [FD PMC Official].
Training	*…“we just step in and out and give them the lead and support and run a demonstration or a workshop”* [Intermediary Agency A, NGO, Green Schools Management Official].
**Maintenance**	
Integration into existing structures	*...“we would have healthy eating in place for years…there was a survey done and the parents wanted a healthy eating policy*” [FD School Coordinator / Primary School Teacher]

#### Implementation

Fidelity to the core components of FD was assessed by project managers employed by the PMC to check the implementation of the programme and was identified as an important factor influencing the success of the programme. Incentives (e.g. stickers, certificates of achievement), which encouraged children to taste and then eat fruit or vegetables were a core component of the FD delivery mechanism.

Adaptations to FD were seen as enabling factors in ensuring successful implementation e.g. adaptations to programme delivery were made for children with special needs and/or serious aversions to specific fruit or vegetables and the timing of the intervention was customised to suit the school timetable.

The school leads delivery of the GST programme. The only core components are the conduct of surveys and walkability audits. Flexibility around implementation is a key feature of the programme. Examples of adaptation include the GS committee composition and conduct and adapting Walking on Wednesdays (WOW) to another day of the week or mode of transport Cycle on Wednesdays, (COW). The programme was deemed to be most successful when children were centrally involved in the different initiatives and competitions across the school. Inter-class competitions e.g. the Golden Boot award and materials e.g. stickers provided by the intermediary, act as incentives for students to participate in the programme.

#### Maintenance/transferability

Dissemination of information through school websites, newsletters, posters, social media, assembly meetings, events and action days and an inter-school symposium to disseminate peer learning were considered important factors aiding success of the GST programme.

Whether continued funding was secured was a key factor in ensuring sustainability of either programme. Another significant factor effecting sustainability was whether or not the programme was embedded in an organisational structure which offered support to the programme e.g. pre-existing healthy eating policies, which reflected the ethos and commitment of the school.

### Factors hindering successful adoption, implementation and maintenance (Table 3)

Insufficient communication between stakeholders at all levels was a perceived barrier e.g. between parents and the schools, within the schools, and between the schools and the programme organisers and funders.

**Table 3:** Quotations illustrating factors hindering adoption, implementation and maintenance of the interventions

(RE)AIM Parameters (Bold)	
**Adoption**	
Collaboration and Communication	*“…the Department insisted that there should be targets… so they asked Green Schools for targets. Green Schools said well just on the basis of programme, this is what we’re getting so far, those targets were extrapolated… Ambitious targets but what the department told them at the time was that you won’t be bound by those targets*” [Intermediary State Agency B Management Official]
Community use	*“…rural schools can be challenging like if there is… a real lack of infrastructure and if they are in a real busy place it can be really challenging…they don’t have as many options open to them, like carpooling can be hard to just kind of get off the ground. You will often have parent concerns about just picking up other people’s children… There is always kind of questions around insurance”* (GSTO).
Training	*“We also ran a cycle course for parents who want to cycle with their children but we didn’t have a very good response there were only two parents who came” [GSTO].*
**Implementation**	
Cultural context	*…“we’ve gone to a situation where no one is walking to school…this is to facilitate working parents”* [Intermediary Agency B, Management Official]
	*“parents are so afraid of roads and road safety… that maybe they are reluctant to let teenagers or their kids out and learn…often it’s just sort of a perceived danger that isn’t really there”* [GSTO].
	*“They (girls) are expected to be on the pink bike going around the park with their pals not out on the road”* [Civil Servant, Government Department B].
	*“It’s uncool yeah” (to wear cycling helmets)* [GSTO].
	*“changes to the norms or family values, that is a big challenge in DEIS (disadvantaged) schools, if you give people fruit and veg in a school and then they go home and their mammies give them a batter burger and chips”* [Civil Servant, Government Department A].
Characteristics of setting	*“We discussed how we were going to get fruit and veg into schools…obviously no school canteen, so it couldn’t go in that way”.* [FD Project Management Company Official].
	*“Our biggest problem is getting the children to come to school, not to talk about how they travel to school.”* (GSTO in socio-economically deprived school catchment area).
Costs, resources and funding needed for implementation	*…“last year was very stressful, like at one point we didn’t know if we would get our funding renewed”* [Intermediary Agency A, NGO, Green Schools Management Official].
Implementation delivery, protocols, fidelity (including dose)	*…“it’s very hard for me to go around and check and see, if they are implementing phase 2 properly … I can’t stand there and say, are you actually checking their lunchboxes and things like that”.* [FD School Coordinator / Primary School Teacher].
Process evaluation	*“(NGO) results would indicate that there is far more walking and cycling than we would think there is in reality”* [Civil Servant, Government Department B].
Time issues	*“We are an infant school so we have a limited number of hours every day in an already overloaded curriculum… that was the concern…”* [FD School Coordinator/ Primary School Teacher]. *“I think a lot of the teachers find it’s long, if it’s a chart it could be nearly a meter long this chart with days and ticking and bronze cert and silver cert and gold certs and all these different certificates and children’s’ names have to be written in”*. [T].
	*“there was quite a bit of timing in all this because you’ve got the school year, you’ve got the calendar year, you’ve got the [name of agency] year, EU funding year and they are all a little bit different so you are trying to manage your money…”* [Civil Servant, Government Department A]
	*…“I should be developing the programme and analysing how we can...make it better, however a lot of my time is spent securing funding” [Intermediary Agency A, NGO, Green Schools Management Official].*
Translation of research into practice	*“We expected to be told, how to implement this programme and it had been researched very thoroughly in the UK…but each research group required a number of PHD students going into the classroom, weighing out the fruit and veg, recording how much they had been eating…So we had a couple of challenges”* [FD PMC Official].
**Maintenance/Sustainability**	
Dissemination and Transferability	*…“We have a difficulty in getting clear data as to what is the actual full impact of the Green Schools programme in a school”* [Civil Servant, Government Department B].
	*“…information relating to ideas that have worked on the ground should be put into hardcopy”* (GSTO).
	*...“the (UK) government had said if you don’t have a school travel plan...you will not qualify for funding for the school, so it’s very easy for a headmaster to say ...we’ll get a school travel plan together. As opposed to our programme which is by invitation only, we’re trying to go in and persuade... there was no comparing like with like.*” [Intermediary Agency B, Management Official]

#### Individual/family level barriers

At individual/family level changing familial norms and eating habits of children were perceived as particularly difficult in the FD programme. In GST parental attitude and busy work schedules were seen as important determinants in the means of transport children use to get to school. The perceived lack of priority around active transport by parents and their reluctance to become involved in other school activities e.g. a cycle training initiative for parents, made implementation of GST challenging. In addition, culturally there was a prolific perception that cycling to school is unsafe and a stereotypical image that cycling, as a means of transport, is unsuitable for girls. Negative peer pressure regarding cycling helmets was also an important barrier.

#### Barriers within the school setting

The time required to implement and record uptake of the programmes was cited as a recurrent challenge. FD was noted to be time consuming to implement in the classroom (cited *10). The time taken to sort out the fruit and vegetables for the FD programme without the aid of additional staff and /or involvement of parents was particularly challenging. The short school day particularly in the infant classes made the delivery of the FD programme difficult in addition to the prescribed curriculum. Timetable and curricular commitments also made implementation of the GST challenging. Different calendars (EU funding year, academic year, calendar year) were challenging from a budgetary and organisational perspective.

The time taken to record activities and monitoring programme implementation was noted to be time consuming and administration dependent. Recording on a wall chart of the portions of fruit and vegetables brought from home was cited as particularly time consuming. The translation of a research based intervention into a national programme with fidelity presented a considerable challenge. In addition, FD coordinators reported it difficult to evaluate the implementation of the FD programme in the classroom. Time spent securing funding was an ongoing barrier for the GST programme.

Accuracy in assessing the impact of the GST programme was a common concern. The absence of a set protocol for GST delivery, differences in the methodological approaches used to set targets and the method used to assess the programme, may have contributed to discrepancies in results. Lack of clear data on effectiveness inhibited dissemination of the programme impact. Moreover, evidence of what worked on the ground was not captured in hardcopy which further inhibited dissemination.

#### Structural and external barriers

The lack of canteens in Irish primary schools presented logistical challenges in terms of organising delivery, packaging and storage of fresh produce in the FD programme. External factors influenced implementation of the GST, in particular poor road and transport infrastructure, concerns around road safety, bicycle safety, security and insurance. The lack of infrastructure near rural schools, though not confined to them, was recognised as a major challenge in terms of promoting active and / sustainable travel.

Absence of a travel plan for the GST, with defined measures, as a requirement to receive government funding, unlike UK and other countries, was cited as significant barrier to long term development and sustainment of GST in Ireland, and made it difficult to compare implementation across countries. Limited funding and/or uncertainty around funding was noted to have a negative impact on programme staff, planning and resource acquisition needed for implementation.

### Supportive actions around adoption, implementation and maintenance (Table 4)

#### Adoption

The provision of support from a variety of internal and external sources was critical to the successful adoption and subsequent implementation of both programmes. In the FD programme, coordinators (teachers) in participating schools were supported by a designated FD Manager, employed by the PMC, who was contactable if any problem arose (cited*2 (Fig. 1a).

**Table 4:** Quotations illustrating supportive actions around adoption, implementation and maintenance of the interventions

(RE)AIM Parameters (Bold))	
**Adoption**	
Collaboration and Communication	*“She (FD Programme Managerl) came and she spoke to me about any concerns, any queries…She was at the end of the phone if there was any issues”.* [FD School Coordinator]
	*…“Some County Councils are very supportive and have provided material resources such as maps which are useful...*” [GSTO].
Training	*…“we developed a course called*” *Get in Gear“... it’s simply just showing them skills it takes to get back on their bike and bring them out for a cycle, and then we evaluate how they feel about cycling after...*” [Intermediary Agency A, Management Official].
**Implementation**	
Characteristics of setting	*…“very dependent on a number of different factors, the delivery on time, the caretaker letting it in, the parents arriving…no problems, as long as the machine was well oiled it was all fine”* [FD School Coordinator].
Implementers’ charcteristics	*…“you will often have a few teachers… who commute in by bike so it’s great to kind of harness in on those*” [GSTO].
Adaptation and customisation	*…“they wanted feedback from the schools…they were told that there was water in the pineapple bag or the cucumber bag… it puts them off the food completely….”*[FD School Coordinator].
	*“If there is a child who has never tasted this or would never eat it in a millions years at home then you have to adapt it to the child involved. You kind of say ‘Right just eat one piece’ or ‘Taste it”* [FD School Coordinator].
	*…“If it is a rural school and they don’t have footpaths or infrastructure then you might be looking at carpooling or maybe park and stride*” [GSTO].
Costs, resources and funding needed for implementation	*…“we’ve got a load of resources…which we have developed over the years, based on problems we’ve faced (and) schools have faced and we’ve come up with an option for them, a solution*” [Intermediary Agency A, Management Official].

The support offered by the GSTO to the school, and by local enterprise including involvement of the police in traffic workshops in the school, were viewed as supportive. In addition, the expertise of the GSTO in facilitating and supporting schools’ actions plans was important. Provision of cycle training for parents in particular was found to encourage a better parental attitude towards cycling with their children.

#### Implementation

The delivery of the FD programme in participating schools was aided by the presence of supportive teaching staff willing to engage in the programme and presence of non-teaching staff willing to assist with its delivery. Constructive feedback had led to better packaging of fruit and vegetables. In addition, allowing teachers' discretion to adapt the programme to suit individual children was perceived as strongly supportive (cited*3). Factors in the external environment e.g. timely delivery of fruit and vegetables by suppliers, was also regarded as a supportive measure.

In the GST Programme, the presence of teachers who acted as clear role models e.g. by cycling to school, was a strong supportive factor.

#### Transfer-dissemination

Development of an internal database of particular queries in the GST programme facilitated peer learning and sharing of ideas between GSTOs.

### Recommendations from stakeholders to overcome barriers (Table 5)

#### Adoption

Personal interest, leadership, commitment, experience of the teacher and principal, and openness to innovation were key factors in overcoming barriers for both programmes. In the case of GST, better communication and collaboration between the NGO and the local authorities was recommended.

**Table 5:** Quotations illustrating recommendations to overcome barriers

(RE)AIM Parameters (Bold)	
**Adoption**	
Collaboration and Communication	*“maybe if we had like a sit down every quarter or whatever and just talk through the issues”* [GSTO].
**Implementation**	
Training	*…“they (boys) had to have their cycle training, they had to disclose it to myself, they had to do their bike maintenance, they had to wear a helmet and they had to have permission from their parents …their cycling numbers went up significantly”* [Green Schools Coordinator (Teacher)].
**Maintenance/Sustainability**	
Cost reduction, other	*“…it proved much easier to sustain a budget, a net budget of XXX.”* [Government Department A, Civil Servant]
	*…“from the publication of the focused policy assessment there was a question whether we should just close the programme…but I think the view from the Department would be that it is a good programme”* [Government Department B, Civil Servant].

### Implementation

Because implementation of GST depended on the practical infrastructure and location of the school, effective traffic management infrastructure at the school gate and provision of cycle lanes were cited as priority areas which needed to be addressed.

Students were disappointed that the green flags awarded for each theme of the Green School Programme were identical. A different emblem for each theme was recommended by stakeholders. Maintaining interest from pupils was an ongoing issue and introduction of short-term projects was suggested as a way to sustain student interest. The organisation of bike maintenance workshops with experts sourced from the local community had resulted in an increase in the number of boys cycling to school and continuation of this activity was recommended.

A recommendation was made to conduct the “Hands Up” surveys on non-event days to enhance the validity of evaluation component of the GST programme. In addition, stakeholders recommended that evaluation be conducted by an independent observer on a periodic basis.

### Sustainability

A number of measures were recommended by stakeholders to inform future development of the programmes. The presence of pre-existing whole school policies were a key factor influencing the sustainability of both programmes. A whole school approach, pre-existing environmental and school safety policies, the long-term continuation of initiatives (e.g. a walking bus) and incentives (e.g. the ‘Golden Boot’ class awards) following the award of the Green Flag were recognized as promoting sustainability of GST. Parental involvement was perceived as key for long-term success of the walking buses. Long term sustainability of FD was aided by a variety of pre-existing healthy eating measures (cited*2) and parental support.

Continued funding was seen as core for sustainability of both programmes. In spite of the recent economic recession funding for FD and GST has continued. A budgetary reduction for the FD, was noted to increase the likelihood of sustainment in the long-term. Ongoing political support was recognised as important for the continuation of the GST despite doubts regarding its overall impact.

## Discussion

This study qualitatively examined the implementation of two school-based obesity prevention interventions. The interventions have been rolled out in other countries, are theory-based and have been previously evaluated. FD is based on well-funded external research evidence; it has clearly defined core components, covers primary schools only and has an uptake of 95% nationally. In contrast GST’s core components are broad rather than specific, implementation is flexible, targets are unclear and it is open to both primary and secondary schools with a lower national coverage. Both programmes are coordinated by external parties, who provide training and technical support, but are delivered by class teachers. Evaluation of FD is precise (e.g. consumption takes place in the classroom) whereas GST has less clear targets and uses a hands-up honesty approach. Key success factors and enablers were identified for both programmes.

Previous research in this area is limited to a recent cross-sectional study of primary schools in Malaysia [19] and to studies conducted with staff in schools with different target age groups, 9–13 year-olds (middle schools) in the US [16, 17] and to teenagers in the Netherlands [18].

Some general themes emerged from our research, which were consistent with these studies. Sufficient communication and collaboration between teachers has been noted previously as being a faciliatory factor [18]. However previous qualitative research has sought only the opinions of school personnel [16–18]. In our study good working relationships and adequate communication at national and local levels, e.g. political backing with associated national and local funding, were identified as key strategies for successful implementation of both programmes.

Involvement of all the partners in the school was critical, from leadership by principals, enthusiastic participation of teachers who led by example ‘champions’, through to student and parent engagement. This commitment by the schools, and buy in from staff members and students has also been noted previously as a key facilitator [16–19]. Presence of an in-school ‘champion’ was one of the strongest reported facilitators to organisational readiness to adopt and sustain the intervention in the Massachusetts Childhood Obesity Demonstration Project in two low income districts, which appeared to offset implementation barriers [16]. Similar to our study the organisational ability of coordinators in making the programmes feasible was also seen as an important factor for implementation success [18].

The programmes chosen for our study had established partnerships with external organisations who provided training, on going support and resources for the schools. This facilitating factor has been noted in a previous study [18] and suggested to be important for on-going sustainability [17].

Within the school the use of incentives to encourage children such as stickers in FD and the awarding of a school flag in GST were enablers. The use of incentives directed at children rather than at teachers has not been reported. Freedom to adapt the interventions to context e.g. for special needs children while maintaining the core components, was viewed as an important enabling strategy. Flexibility in terms of programme delivery was also noted as an enabler in the Dutch study [18]. On-going adaptation of interventions with a primary focus on fit between the intervention and multi-level contexts lead to expectation of on-going improvement of interventions rather than their diminution over time [36]. However, while aspects of the flexibility of the GST programme were welcomed it also created difficulties for some of the participants. Defining success in the GST programme was particularly problematic and difficulties focused on poor communication around clear targets and subjective evaluation (hands-up) methods.

Barriers associated with budgetary limitations [17] and time constraints [17, 19] have previously been noted. Lack of time to implement and record obesity prevention interventions during the daytime curriculum, particularly in infant classes, was a particular issue for our study. Funding insecurity, particularly for GST, led to a feeling of uncertainty although this was a challenge to long-term sustainability of both programmes. Other barriers identified in these studies; high staff turnover [16, 18], lack of equipment [19] and insufficient or lack of access to training [19, 37] were not applicable to our setting.

Existing attitudes and perceptions of the target action acted as a considerable barrier to greater levels of engagement. These were associated with safety and social pressure i.e. perception of cycling as being inappropriate for girls. Some of these beliefs may be well founded particularly in implementing an intervention where the broader infrastructure was insufficient. Engaging pupils and especially parents of disadvantaged pupils was particularly problematic and required a deep understanding by teachers of the context of the children’s home lives where fundamental issues such as school attendance rather than anti-obesity interventions were the top priority.

A strength of our study is that we interviewed stakeholders involved in the funding and organisation of the programmes in addition to school staff, thus bringing a wider perspective to the factors involved in implementation of school-based obesity prevention programmes. Engaging participation of key stakeholders was essential and all stakeholders invited to participate agreed to be interviewed. As the researcher who conducted the interviews was not known to the stakeholders, this minimised the liklihood of socially desirable responses.

However the pragmatic nature of this study means that it has some limitations. Non-randomisation of FD schools creates the possibility of bias towards the selection of schools where the programme was more successfully implemented. However, issues of concern around implementation were identified for both programmes.

A whole school approach involving pupils, teachers and parents is deemed necessary for sustained impact of lifestyle interventions in schools [38]. However, only the opinions of teachers were sought in this study, reducing the comprehensiveness of the findings.

Research on the “health promoting schools” initiative has recommended that process evaluation must move beyond simple measures of acceptability and fidelity to include detailed contextual information [39]. Good contextual fit occurs when implementers, recipients, and other stakeholders (e.g. parents, teachers), identify an intervention as acceptable, doable, effective, and sustainable in their local setting.

Our findings provide insight into the facilitators and barriers that must be addressed to successfully implement health behaviour change interventions in the school setting and provide guidance in setting out parameters for evaluation to include fidelity and adaptation. This pragmatic approach also provides recommendations on how identified barriers may be overcome/reduced. Although some recommendations are particular to the school setting and relate to shaping the future of these programmes, many are generalisable across different contexts. The findings are highly relevant to those involved in the organisation and execution of national public health interventions; policy makers, guidelines developers, those involved in the local organisation of the interventions and frontline staff involved in their delivery.

Implementation of complex public health interventions requires leadership and commitment at and across all levels. Stronger alliances between health and education professionals and the impact of future interventions must address health and educational outcomes to improve scalability [39]. The absorptive capacity of teaching staff to engage with and deliver behavioural interventions is a constant challenge in an overcrowded curriculum. An unanswered question for future research is the concept of reaching a “tipping point” when attitudes of staff harmonise to support a particular intervention/policy.

## Conclusion

Successful implementation and scale up of public health anti-obesity interventions in schools is dependent on having a good contextual fit between the intervention and setting, engagement and leadership at multiple levels, and secure funding. Pre-existing healthy lifestyle policies within a school were a strong supportive factor. At individual level, tailoring the intervention and use of incentives were important enablers. Recommendations to overcome barriers include: capacity to deliver within an already overcrowded curriculum, supports needed to do so, and clear specification of intervention components within a conceptual framework to facilitate evaluation. The importance of adaptation without compromising core components in enhancing intervention sustainability also emerged. Our findings are generalisable across different contexts and are highly relevant to those involved in the development or adaptation, organisation or execution of national public health interventions in the schools setting; policy makers, guidelines developers, and staff involved in local organisation and delivery of such interventions.

## Additional file

Additional file 1: Topic Guide; Open questions to stakeholders. (PDF 124 kb)

## Abbreviations

BMI: Body Mass Index
DEDIPAC-KH: Determinants of Diet and Physical Activity Knowledge Hub
EU: European Union
FD: Food Dudes
GSP: Green Schools Programme
GST: Green Schools Travel
GSTOs: Green School Travel Officers
JPI-HDHL: Joint Programming Initiative-Healthy Diet for a Healthy Life
NGO: Non-Governmental Organisation
PMC: Project Management Company
RE-AIM: Reach, Effectiveness, Adoption, Implementation, Maintenance
