# Supplementary material for: Barriers and facilitators to adoption, implementation and sustainment of obesity prevention interventions in schoolchildren– a DEDIPAC case study
Source: BMC Public Health. 2019 Feb 15;19:198. doi: 10.1186/s12889-018-6368-7 (PMC6377757; doi:10.1186/s12889-018-6368-7)
Supplement: Supplementary file 1 — Topic Guide; Open questions to stakeholders. (PDF 124 kb) [file 12889_2018_6368_MOESM1_ESM.pdf]

Open questions

- "From your point of view, which factors contributed to a successful implementation of the [XYZ] intervention?"
- "Could you please describe these factors in further detail?"
- "From your point of view, which factors hindered/ inhibited/ slowed down the implementation of the [XYZ] intervention?"
- "Could you please describe these factors in further detail?"
- "From your point of view, how can these factors/ issues be addressed? How can possible barriers be overcome?"
- "From your point of view, what made this specific intervention sustainable after the implementation phase was completed? If it was not sustained, what were the reasons for the lack of sustainability?"

Prompts

If the interviewee does not provide information regarding all factors listed below, you may use the prompts to elicit further information. Please keep in mind that you do not have to ask all of these questions. You may ask about the topics that have not been addressed in response to the open questions stated above. Please do not read out the headings in square brackets to the interviewee.

**Prompts**	**Already discussed**	**Prompted**
**1. ADOPTION**		
**[Training for implementation]**<br><br>"Was the implementation staff trained sufficiently for the adoption of the intervention (e.g., certificate training, workshops, training instructions, regular meetings/ supervision, technical assistance)?"	○	○
**[Staff expertise for implementation]**<br><br>"Do you think that the program staff's skills, knowledge and competence were adequate to implement the program?"	○	○
**[Community use]**<br><br>"Was local community involvement secured (e.g. use of community organizations, relationships/ networks between implementing organizations and community organizations)?"	○	○
**[Collaboration and communication]**<br><br>"Were multiple stakeholders involved in the program implementation (stakeholders from health and sport sectors, also food, transportation, planning and housing, green	○	○

spaces, education, healthcare, social services)? How did implementers and stakeholders communicate? Which methods were used to facilitate the communication process?”		
**2. IMPLEMENTATION**		
**[Theory use]**<br><br>“Was implementation theory used to facilitate the implementation of the intervention (e.g., the RE-AIM model)? If yes, explain how? From your point of view, did the use of theory contribute to a successful implementation? If yes, how? If not, why not? If implementation theory was not used, could you please explain why this was not done?”	○	○
**[Delivery characteristics: dose & fidelity]**<br><br>“Was intervention dose and fidelity monitored with an implementation protocol? In other words, did you monitor how much of the intervention was delivered and how close the intervention activities were to the intervention protocol? If yes, how was this protocol used? From your point of view, did the monitoring contribute to a successful implementation? If yes, how? If not, why not? If no protocol was used, what was done instead to ensure intervention fidelity?”	○	○
**[Adjustments and customizations]**<br><br>“Was the intervention tailored to<br><br>a.) the target population (e.g., socio-cultural background, gender, participants’ needs),<br><br>b.) local conditions or the setting,<br><br>c.) expertise of the staff responsible for implementing the intervention.<br><br>If any of the options are answered with a yes: Please describe in further detail how the intervention was tailored? Could you please explain what worked well with regards to the tailoring? What worked less well?”	○	○
**[Simplicity of the intervention]**<br><br>“From your point of view, was the intervention easy to follow? Were the components easy to understand? If yes, how did the simplicity of the intervention facilitate participation? If not,	○	○

please elaborate how a lack of simplicity may have affected implementation?"		
**[Accessibility]**<br><br>"Was the intervention accessible to participants? Did the implementation conditions facilitate/ hinder the implementation (e.g., Was participation in the intervention free or if not, was participation affordable? Did the physical environment support the intervention goals, e.g., if an intervention goal was regular walking, a feature of the physical environment enhancing this behavior could be the availability of footpaths, stairs)? If yes, how? If not, why not?"	○	○
**[Time issues]**<br><br>"Were there time issues affecting the implementation of the intervention? If yes, could you describe in detail which issues affected the implementation and how (e.g., lack of time in the community involved/among intervention staff to implement intervention/among intervention participants)? If there were no issues, could tell us about the time schedule for the implementation?"	○	○
**[Cultural context]**<br><br>"From your point of view, did the intervention address specific cultural characteristics of the target population? If yes, how? If not, why not?"	○	○
**[Costs and funding/ resources needed for delivery]**<br><br>"Was sufficient funding provided for the implementation and maintenance of the intervention (in terms of staff, materials)? If yes, could you please give more details on the funding? If not, why was it insufficient? Which problems occurred?"	○	○
**[Characteristics of the setting affecting delivery/ implementation]**<br><br>"Were there any characteristics of the setting that affected the intervention implementation? If yes, which ones, and how did they affect the implementation (e.g., organizational practices/ culture, policies)?"	○	○
**[Implementers' characteristics affecting implementation]**	○	○

"How did characteristics of the implementers affect the implementation (e.g., expectations/ motivation/ perceived control regarding the process of the implementation)?"		
**[Implementations process evaluation]**<br>"Was a process evaluation conducted? If yes, which factors were assessed and documented, and how long was the follow-up period? If not, please explain why not?"	○	○
**3. MAINTENANCE**		
**[Factors contributing to sustainability]**<br>"Was there an institutionalization of the program (e.g. integration into existing institutional programs)? If yes, how was it integrated? If not, why not? Which strategies were used to promote long-term participation? Which of them were more successful and which ones were less successful?"	○	○
**4. TRANSFER**		
**[Dissemination]**<br>"Which actions were taken to disseminate the program? Which channels were used for dissemination? (e.g., media, informal channels)? What worked/ did not work?"	○	○
**[Staff and stakeholders: Training for transfer]**<br>"Was the implementation staff trained in the cultural adaptation of such programs?"	○	○
**[Differences in health care systems across countries]**<br>"If the program was disseminated in various countries, were differences in health care systems taken into account?"	○	○
